# Descriptions of a new Brazilian *Tacora* species and the female of *Tacora saturata*, and a key to the species of the genus (Insecta, Hemiptera, Cicadellidae, Cicadellini)

**DOI:** 10.3897/zookeys.160.2080

**Published:** 2011-12-29

**Authors:** Gabriel Mejdalani, Roberta Santos Silva, Cláudia Garcia

**Affiliations:** 1 Departamento de Entomologia, Museu Nacional, Universidade Federal do Rio de Janeiro, Quinta da Boa Vista, São Cristóvão, 20940–040, Rio de Janeiro, RJ, Brasil

**Keywords:** Auchenorrhyncha, Cicadellinae, identification key, leafhopper, taxonomy, Venezuela

## Abstract

*Tacora johanni*, a new species from Rondônia State, North Brazil, is described and illustrated. The new species can be recognized by the male genital features, especially the subgenital plates with the basal half distinctly expanded and with outer lateral margin round, the long and slender preapical pygofer process, and the styles with apical half strongly curved. Also, the genus is recorded for the first time from Venezuela, based on specimens of *Tacora saturata* Young, 1977, while the female of this species (here described in detail for the first time) shows two unusual features of the genitalia. A key to males of all known *Tacora* species and a map showing the known distribution of the genus are provided.

## Introduction

The South American genus *Tacora* Melichar, 1926 is known from five species (Young 1977, Takiya and Mejdalani 2002, McKamey 2007): *Tacora dilecta* (Walker, 1851) (type species), *Tacora saturata* Young, 1977, *Tacora cavichiolii* Takiya & Mejdalani, 2002,  *Tacora henriquesi* Takiya & Mejdalani, 2002, and *Tacora karipuna* Takiya & Mejdalani, 2002. This genus was recorded from Brazil (Acre, Amazonas, Mato Grosso, and Rondônia states), Colombia, and Peru, being restricted to the central and western portions of the Amazon region (Takiya and Mejdalani 2002). Among the Neotropical Cicadellini, *Tacora* species can be recognized by their distinctive color pattern: body yellow or orange with at least two conspicuous brown stripes on the forewings, one on clavus along the claval sulcus, the other transverse, transcommissural, located in the apical portion of clavus and extending over the corium towards the costal margin. Additional black, brown, red, lilac, or yellow markings are also present and vary interspecifically. According to a morphological cladistic analysis (Cavichioli 1992) of the 13 genera of the *Paromenia* group (established by Young 1977), *Tacora* is the sister group of *Dasmeusa* Melichar, 1926. The reader is referred to Takiya and Mejdalani (2002) for detailed comments on the taxonomy, phylogeny, and distribution of *Tacora*.

In the present paper, we describe a new *Tacora* species from Rondônia State (North Brazil). We also provide the first detailed description and illustrations of the female of *Tacora saturata*, including two unusual features of the genitalia. The genus is recorded from Venezuela (Amazonas and Bolivar states) for the first time (based on *Tacora saturata*). A new key to males of *Tacora* species and a map showing the known distribution of the genus, both modified from Takiya and Mejdalani (2002), are provided.

## Material and methods

Techniques for preparation of male and female genital structures follow Oman (1949) and Mejdalani (1998), respectively. The dissected genital parts are stored in microvials with glycerin and attached below the specimens, as suggested by Young and Beirne (1958). The descriptive terminology adopted herein follows mainly Young (1977), except for the facial areas of the head (Hamilton 1981, Mejdalani 1998) and the female genitalia (Nielson 1965, Hill 1970). Mejdalani (1998) provided a detailed justification for the use of Hamilton’s terminology for the head of the Cicadellinae and other leafhoppers. Use of the term gonoplac (= third ovipositor valvula) and the names for the processes of the dorsal and ventral sculptured areas of the first ovipositor valvula follow Mejdalani (1998).

Label data are given inside quotation marks with a reversed virgule (\) separating lines on the labels and a semicolon separating labels of a specimen. The specimens studied belong to the following institutions: American Museum of Natural History (AMNH; New York), Museu Nacional, Universidade Federal do Rio de Janeiro (MNRJ; Rio de Janeiro), and Museo del Instituto de Zoología Agrícola, Universidad Central de Venezuela (MIZA; Maracay).

The photographs of the body in dorsal view and of the general lateral views of the ovipositor valvulae were prepared with the software Automontage (Synoptics Inc., Frederick, Maryland) using a digital camera attached to a stereomicroscope. The photographs of the details of the ovipositor valvulae were taken with a digital camera attached to an optical microscope. Digital images of four of the five previously known *Tacora* species (body in dorsal view) are available in the internet site “Sharpshooter Leafhoppers of the World” (Wilson et al. 2009). These images were useful for the comparisons and identifications carried out in the present study. The orientation of the illustrations of male and female genital structures on the plates are in accordance with the monograph of Young (1977).

## Results

### Genus Tacora Melichar, 1926

#### Tacora johanni sp. n.

urn:lsid:zoobank.org:act:4E10B39B-9F6B-42EC-B9A7-21B25D2AA4FE

http://species-id.net/wiki/Tacora_johanni

Figs 1a 2 3 

##### Description of the male holotype.

Length 15 mm from apex of head to apex of forewings at rest. Head (Fig. 2a, b) strongly produced anteriorly; median length of crown approximately 4/5 interocular width and 1/2 transocular width; anterior margin broadly rounded in dorsal view; without carina at transition from crown to face; ocelli located behind imaginary line between anterior eye angles, each ocellus slightly closer to median line of crown than to adjacent anterior eye angle; crown with concavity between eye and ocellus, without median fovea and without sculpturing or setae; frontogenal sutures extending onto crown and attaining ocelli; antennal ledges, in dorsal view, slightly protuberant, in lateral view with anterior margin broad and rounded; frons convex, muscle impressions distinct; epistomal suture obscure; clypeus not produced, its contour continuing profile of lower portion of frons.

Thorax (Fig. 2a, b) with pronotal width distinctly greater than transocular width; lateral pronotal margins convergent anteriorly; posterior margin slightly concave; dorsopleural carinae complete, rectilinear, distinctly declivous anteriorly; pronotal disk without sculpturing or pubescence; mesonotum with scutellum not swollen. Forewings (Fig. 2c) with membrane including all of apical cells except short basal portions of second, third, and fourth; veins mostly indistinct except at wing apical portion; with four apical cells, base of fourth more proximal than base of third (not shown in Fig. 2c); without anteapical plexus of veins. Hindlegs with femoral setal formula 2:1:1; length of first tarsomere greater than combined length of two more distal tarsomeres; plantar surface with two parallel rows of small setae.

**Figure 1.**
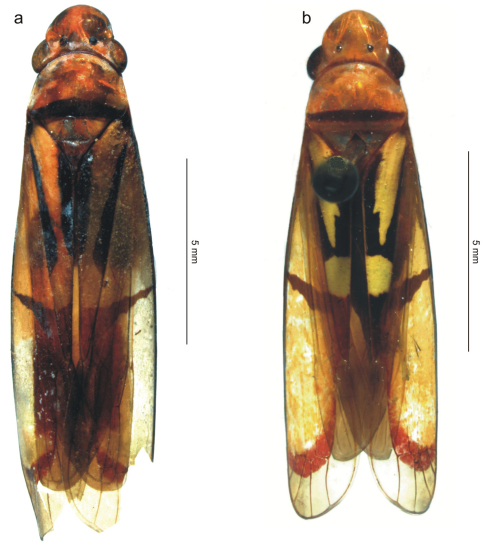
The two species treated in this paper, body in dorsal view (antennae and legs not depicted). **a ***Tacora johanni* sp. n., male holotype **b** *Tacora saturata* Young, 1977, female (pinned specimen).

**Figure 2.**
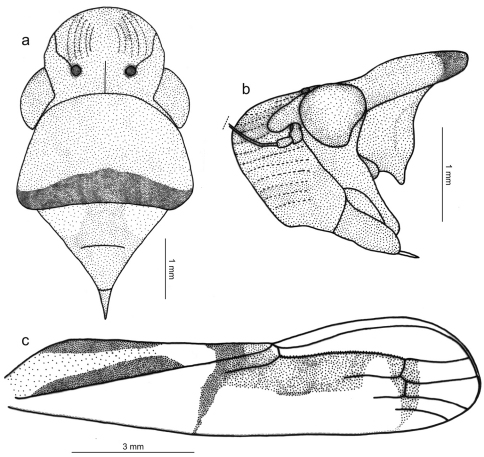
*Tacora johanni* sp. n., male holotype. **a** head, pronotum and mesonotum, dorsal view **b** anterior portion of body, lateral view **c** left forewing.

Color (Fig. 1a) of anterior dorsum orange; pronotum with transverse brown stripe on posterior margin, this stripe distinctly broader medially; mesonotum with pair of large yellow maculae laterobasally. Forewings (Fig. 2c) with corium mostly translucent yellow, costal margin dark brown; clavus mostly yellow with two longitudinal brown stripes, both extending from basal portion to about middle area, one along claval sulcus (broadened posteriorly) and another along inner claval margin; posterior portion of clavus with transverse, oblique brown stripe extending over corium and reaching costal margin, this stripe distinctly narrowed towards costal margin; with red area behind transverse stripe covering apex of clavus, apexes of brachial and inner discal cells and large portion of corium adjacent to inner apical cell, this red area strongly constricted posteriorly and then forming stripe (evanescent towards costal margin) across bases of apical cells; corium region between red area and costal margin mostly depigmented. Face and venter orange-yellow with brown irregular areas on legs.

**Figure 3.**
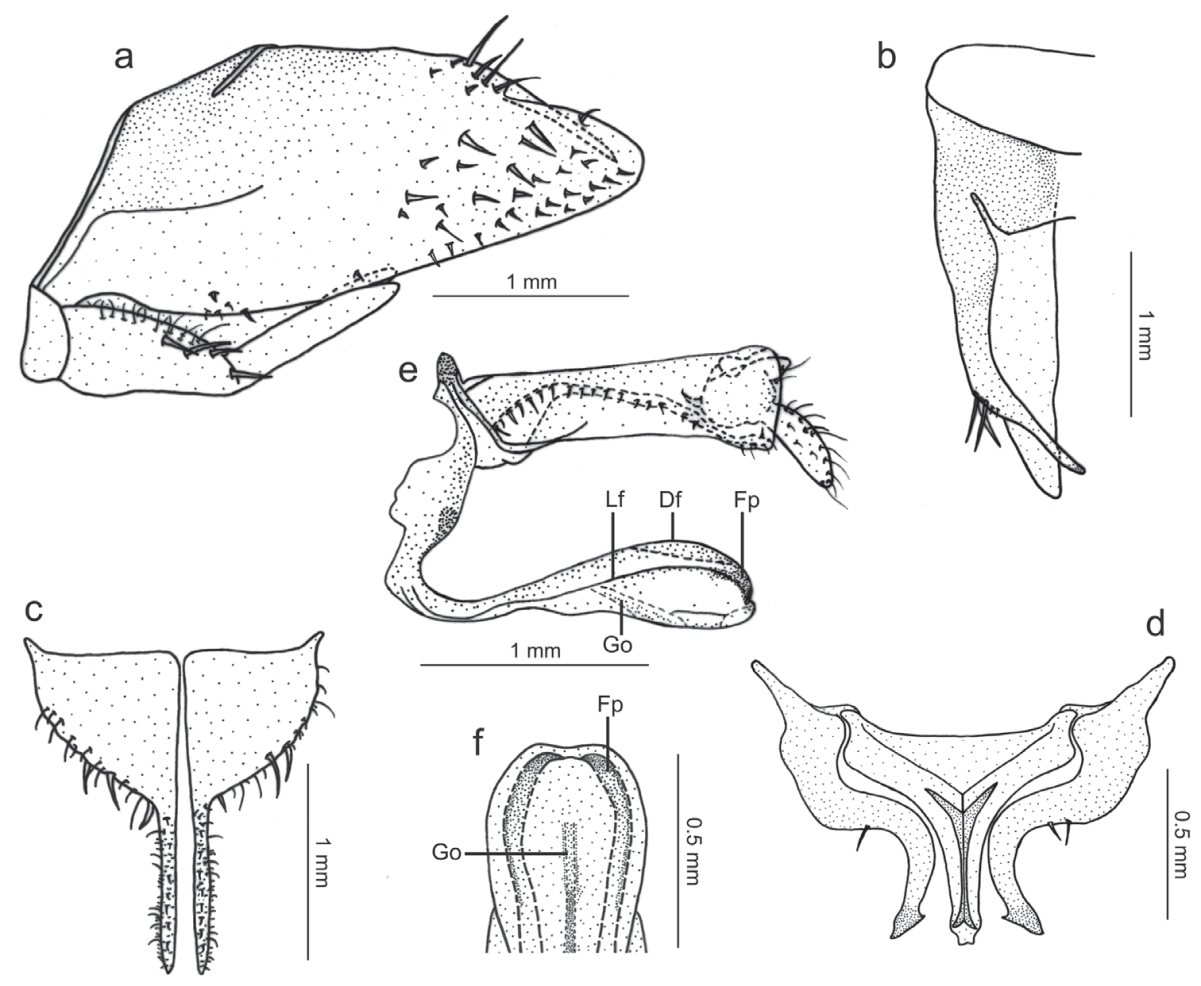
*Tacora johanni* sp. n., genitalia of male holotype. **a** pygofer, valve and subgenital plate, lateral view **b** pygofer, dorsal view **c** subgenital plates, ventral view **d** styles and connective, dorsal view **e** aedeagus and anal tube, lateral view (Df: dorsal flange, Fp: projection formed by flanges, Go: gonoduct, Lf: lateral flange) **f** apical portion of aedeagus, ventral view.

Genitalia with pygofer (Fig. 3a), in lateral view, strongly produced posteriorly; posterior margin narrowly rounded; distal third of dorsal margin with strong spiniform process, apex of process located slightly before pygofer apex, process (Fig. 3b), in dorsal view, directed inward, not attaining median body line; macrosetae distributed mostly on distal third of disk, some smaller macrosetae extending anteriorly along ventral margin. Subgenital plates (Fig. 3c), in ventral view, triangular but with basal half broad, expanded, with lateral margin round; distal half extremely narrow; plates not fused to each other basally; in lateral view, plates distinctly shorter than pygofer (Fig. 3a), extending posteriorly approximately as far as 2/3 of pygofer disk length; plates with uniseriate macrosetae (very large on basal half, very small on distal half), microsetae also present. Connective (Fig. 3d), in dorsal view, broadly T-shaped; stalk with strong median keel, the latter bifurcated basally and extending for short distance over arms. Styles (Fig. 3d), in dorsal view, extending almost as far posteriorly as connective; strongly curved inward; without preapical lobe; apex slightly expanded, obliquely truncate, more sclerotized than remainder of apophysis; style median portion with a few setae on outer margin. Aedeagus (Fig. 3e, f), in lateral view, narrowest in basal third, gradually broader in distal two-thirds; shaft with two pairs of longitudinal flanges, one along lateral portion, another along dorsal margin; apical portion of shaft (Fig. 3f), in ventral view, with these flanges forming pair of projections directed medially; these projections covered by many tiny tegumentary processes; gonoduct distinctly visible in broadened portion of shaft (Fig. 3e), directed ventrally, gonopore located preapically.

**Female.** Unknown.

##### Material examined.

 Male holotype, “BRASIL: Rondônia, \ Ouro Preto d'Oeste [10°40'S, 62°18'W], \ 28.X.1983 \ J. Becker, O. Roppa & \ B. Silva” (MNRJ). The holotype is in good condition but the forewing apices are damaged (Fig. 1a).

##### Etymology.

The new species is described in honor of the late Prof. Johann Becker (Museu Nacional, Universidade Federal do Rio de Janeiro) in recognition of his contribution to the development of Brazilian entomology. He was also one of the collectors of the holotype.

##### Remarks.

**Figure 4.**
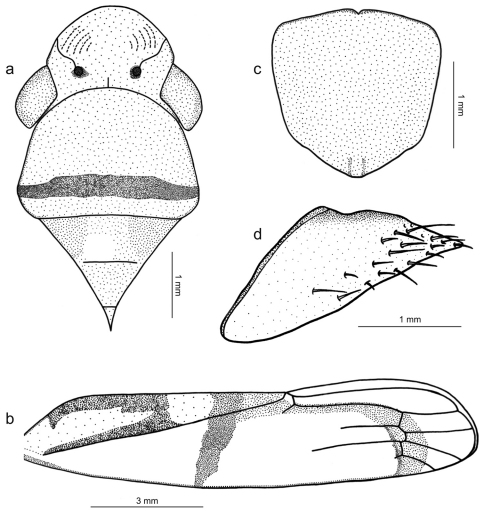
*Tacora saturata* Young, 1977, female. **a** head, pronotum and mesonotum, dorsal view (the white circle on the mesonotum delimits the pin perforation) **b** left forewing **c** sternite VII, ventral view **d** pygofer, lateral view.

*Tacora johanni* can be distinguished from the other known species of the genus by the following combination of features: (1) pronotum with a transverse brown stripe on posterior margin (Figs 1a, 2a); (2) subgenital plates extending posteriorly approximately as far as 2/3 of pygofer disk length (Fig. 3a); (3) subgenital plates with the basal half broad, expanded, with round lateral margin (Fig. 3c); (4) preapical pygofer process long and slender; (5) preapical pygofer process without pegs (Fig. 3a, b); (6) styles strongly curved inward (Fig. 3d); (7) aedeagus with two pairs of longitudinal flanges (Fig. 3e).

The male genitalia of the new species are similar to those of *Tacora dilecta* and *Tacora cavichiolii*. The presence of sculpturing (pegs) on the pygofer process of the male holotype of *Tacora dilecta* was mentioned and illustrated by Young (1977: 317 and Fig. 257p) as a diagnostic feature of this species. These pegs are not present in *Tacora johanni*. Other features that distinguish the new species (known only from the male) from *Tacora dilecta* include (1) the red color of the claval apex behind the transverse dark stripe (orange to yellow in *Tacora dilecta*) and (2) the rectilinear distal portion of the pygofer process in dorsal view (distinctly curved in *Tacora dilecta*). The above-mentioned features of the subgenital plates and styles of *Tacora johanni* are distinct from those of *Tacora dilecta* and *Tacora cavichiolii*. In addition, the pygofer process in *Tacora cavichiolii* is short and robust (Takiya and Mejdalani 2002: 239: Fig. 17), whereas it is long and slender in *Tacora johanni*.

#### Tacora saturata

Young, 1977

http://species-id.net/wiki/Tacora_saturata

Figs 1b 4 5

Tacora saturata  Young, 1977: 316, figure 258 (crown, pronotum, and male genitalia).

##### Description of the female.

 Length 13 mm from apex of head to apex of forewings at rest [male with about same length]; median length of crown approximately 9/10 interocular width and 1/2 transocular width. Head and thorax (Fig. 4a) much as in the above-described male holotype of *Tacora johanni* sp. n. Forewings extending well beyond apex of ovipositor.

Color (Fig. 1b) of anterior dorsum orange; pronotum with transverse, submarginal brown stripe on posterior portion, this stripe broader medially; mesonotum with pair of large brown maculae laterobasally. Forewings (Fig. 4b) with corium mostly translucent orange, costal margin dark brown; in rest position with transcommissural, brown W-shaped figure in basal half of clavi with anterior angles enclosing large, bright yellow maculae that extend to wing bases; large, bright yellow rounded transcommissural macula located just behind W-shaped figure, followed by transverse, oblique brown stripe extending over corium and reaching costal margin; apical portion of clavus red, with or without pair of small orange spots adjacent to claval sulcus just behind transverse brown stripe; distal half of corium with red stripe in inner anteapical cell (along outer margin of inner apical cell) and then descending across bases of apical cells.

**Figure 5.**
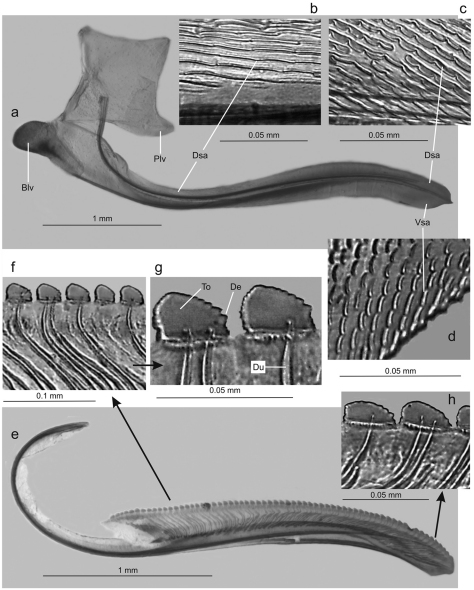
*Tacora saturata* Young, 1977, ovipositor. **a** valvifer I and valvula I, lateral view (Blv: basal lobe of valvula I, Dsa: dorsal sculptured area, Plv: posterior lobe of valvifer I, Vsa: ventral sculptured area) **b** dorsal sculptured area at basal portion **c** dorsal sculptured area at apical portion **d** ventral sculptured area **e** valvula II, lateral view **f-g** teeth at basal portion (De: denticle, Du: duct, To: tooth) **h** teeth at apical portion.

Genitalia with abdominal sternite VII (Fig. 4c) broad, lateral margins convergent posteriorly, posterior margin broadly convex. Internal sternite VIII without distinct, well-defined sclerotized areas. Pygofer (Fig. 4d), in lateral view, well produced posteriorly, strongly narrowed towards apex; posterior margin very narrow, subacute; macrosetae located mostly on distal third, a few extending anteriorly along ventral margin. Valvifers I (Fig. 5a), in lateral view, of quadrate form, except for distinct lobe on posteroventral portion. Valvulae I, in ventral view, distinctly expanded basally; in lateral view (Fig. 5a), valvulae with broad basal lobe, blade distinctly expanded in distal half in comparison with basal half; dorsal sculptured area (mostly scale-like processes, except for linear processes basally, Fig. 5b, c) extending from basal portion of blade to apex, ventral sculptured area (scale-like processes, Fig. 5d) restricted to apical portion of blade; basal portion of blade with group of distinct setae located below ramus; apex of blade forming distinct dentiform projection (Fig. 5a); ventral interlocking device distinct, elongate, restricted to basal half of blade, located along ventral blade margin but with distal portion directed dorsally. Valvulae II (Fig. 5e), in lateral view, with dorsal margin regularly convex beyond basal curvature; without preapical prominence; apex obtuse; ventral outline of apical portion slightly concave; about 63 teeth (Fig. 5e, f, g, h), mostly subtriangular or subrectangular, distributed on dorsal portion of blade, with clear space between them; denticles (Fig. 5g) on posterior portions of teeth and on inferior half of apical portion; blade with numerous curved ducts (Fig. 5f, g) extending to teeth or terminating below the latter, ducts also extending towards apex (most teeth receive a single duct, others two or none). Gonoplacs, in lateral view, extending posteriorly slightly beyond pygofer apex; basal half with ventral margin convex and dorsal margin concave, the latter abruptly expanded towards distal half; ventral margin of distal half concave; apex of blade rounded; apical portion with few small setae and tiny tegumentary processes, the latter extending anteriorly along ventral margin.

##### Material examined.

 One male and one female, “Venezuela, T. F \ Amazonas Dpt \ Rio Negro”; “Rio Baria \ 140m. \ 0°55'N, 66°10'W”; “C. Padilla \ col. \ 28-II-84” (MIZA); one male, same as preceding, excepting “12-II-84” (MIZA); one male, same as preceding, excepting “7-III-84” (MIZA); one male, same as preceding, excepting “4-III-84” (MNRJ); one female, same as preceding, excepting “20-II-84” (MIZA); one male and one female, same as preceding, excepting “L. J. Joly \ A. Chacon \ 4–11-II-84” (MIZA); one male, same as preceding, excepting “Rio Mawari- \ numa 140m”; “3-III-84 \ C. Padilla \ col.” (MIZA); one female, “Venezuela T. F. \ Amazonas. \ 25-XI-4-XII-1984”; “Rio Baria \ 140m. \ 0°55'N, 66°10'W”; “E. Osuna \ A. Chacón” (MNRJ); three males and one female, “VENEZUELA: Amazonas \ Cerro Unturan Camp, 65°14'W \ 01°33N, 1100m. 11–15/III/89”; “Phipps-FUDECI Exped. \ by Amer. Mus. Nat. Hist. \ D. A. Grimaldi, coll.” (AMNH); one female, “Venezuela - Boli- \ var Rio Caura. \ 26-IV-1984”; “Salto Pará” [06°12'N, 64°28'W]; “B. Bechyne. \ leg.” (MIZA).

##### Remarks.

Young (1977) provided a detailed description of the male of *Tacora saturata*. Our identification is based on his description. The male genitalia and color pattern (male and female) of our Venezuelan specimens agree with the features and illustrations given by him. He recorded *Tacora saturata* from Colombia and Brazil. This species is the only one in the genus in which the transverse pronotal stripe is submarginal (Figs 1b, 4a).

### Key to males of Tacora Melichar (modified from Takiya and Mejdalani 2002 to include Tacora johanni sp. n.)

**Table:** 

1a	Pronotum with posterior submarginal transverse stripe (Figs 1b, 4a; observed also in the female); subgenital plates extended posteriorly beyond pygofer apex (Young 1977: Fig. 258c)	*Tacora saturata* Young, 1977
1b	Pronotum with transverse stripe on posterior margin (Figs 1a, 2a); subgenital plates not extended posteriorly beyond pygofer apex.	2
2a	Forewing corium with lilac band along claval sulcus basad of transverse brown band (observed also in the female); pygofer about five times longer than width on median portion in lateral view, dorsal preapical processes with short dentiform projection on inner margin in dorsal view.	*Tacora henriquesi* Takiya & Mejdalani, 2002
2b	Forewing with corium region along claval sulcus concolorous yellow or orange basad of transverse brown band (Fig. 1a, b); pygofer not more than three times longer than width on median portion in lateral view, dorsal preapical processes without conspicuous projection.	3
3a	Pygofer preapical dorsal processes bearing pegs on apical portion (Young 1977: Fig. 257p); aedeagus without flanges or processes (Young 1977: Fig. 257f)	*Tacora dilecta* (Walker, 1851)
3b	Pygofer preapical dorsal processes lacking sculpturing; aedeagus with dorsal and lateral carinate flanges.	4
4a	Aedeagus with four pairs of dorsolateral flanges, apical flanges very short and triangular in ventral view	*Tacora karipuna* Takiya & Mejdalani, 2002
4b	Aedeagus with two pairs of lateral flanges	5
5a	Subgenital plates with basal half distinctly expanded, with round outer lateral margin (Fig. 3c); preapical pygofer process long and slender (Fig. 3a, b); styles with apical half strongly curved (Fig. 3d)	*Tacora johanni* sp. n.
5b	Subgenital plates with basal half not expanded, with outer lateral margin more rectilinear, narrowing gradually towards median portion; preapical pygofer process short and robust; styles with apical half slightly curved.	*Tacora cavichiolii* Takiya & Mejdalani, 2002

## Concluding remarks

Six species of *Tacora* are now known. The most relevant features for the recognition of males from each of these species are given in our key, which provides an adequate comparison among the previously described species and *Tacora johanni* sp. n. These features are from the aedeagus and its flanges, pygofer and its processes, subgenital plates and styles, as well as from the color pattern.

Females of *Tacora* are of difficult identification when they can not be associated to males from the same collecting series, due perhaps to our still incomplete comparative knowledge of the female genitalia morphology and color pattern intraspecific variation. In addition to our description of *Tacora saturata*, the female genitalia of *Tacora dilecta*, *Tacora henriquesi*, and *Tacora cavichiolii* were described in detail (the first by Young 1977 and the second and third by Takiya and Mejdalani 2002). Our study of the *Tacora saturata* female revealed peculiar, previously undescribed genital features of potential taxonomic interest that deserve further comparative investigation, i.e. the lobe on the posteroventral portion of valvifer I and the dentiform projection on the apex of valvula I (Fig. 5a). Unfortunately, these parts of the female genitalia were not described for the other species.

**Figure 6.**
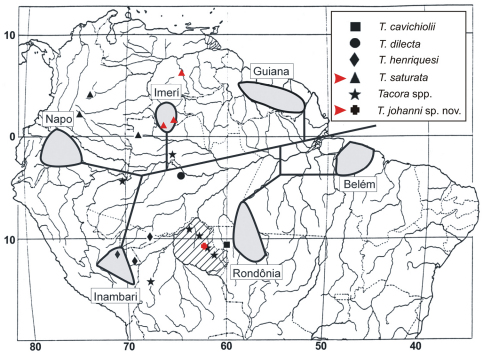
Distributional map of collecting sites of *Tacora* species (modified from Takiya and Mejdalani 2002). Records established in the present study are in red on the map. *Tacora* spp.: unidentified females. Rondônia State appears hatched due to the uncertain type locality of *Tacora karipuna*. Grey areas connected by black lines correspond to the central portions of six areas of endemism and their possible historical relationships (see details and additional references in Takiya and Mejdalani 2002).

The previously known distribution of *Tacora* (Brazil, Colombia, and Peru) is now extended to Venezuela (Fig. 6). Takiya and Mejdalani (2002) suggested that a vicariant event isolated the *Tacora* ancestor in Western Amazonia in an area that included the Napo and Inambari endemism centers, and possibly also the Imerí center (where no records of the genus were known at that time). Interestingly, the present results appear to support the proposal of Takiya and Mejdalani (2002), as we found that *Tacora saturata* is distributed in the Imerí center (Fig. 6).

## Supplementary Material

XML Treatment for Tacora johanni

XML Treatment for Tacora saturata
